# Isolation, Identification, and Susceptibility Profile of *E. coli*, *Salmonella*, and *S. aureus* in Dairy Farm and Their Public Health Implication in Central Ethiopia

**DOI:** 10.1155/2022/1887977

**Published:** 2022-02-14

**Authors:** Umer Seid Geletu, Munera Ahmednur Usmael, Abdulmuen Mohammed Ibrahim

**Affiliations:** ^1^Collage of Agriculture, Animal Science Department Oda Bultum University, P.O. Box 226, Chiro, Ethiopia; ^2^West Hararghe Zone, Chiro Wereda, Animal Health Worker, P.O. Box 226, Chiro, Ethiopia; ^3^Regional Project Manager of L Caged Project, Haramaya University and University of Florida, Gainesville, FL, USA

## Abstract

A cross-sectional study was conducted from November 2018 to May 2019 in Bishoftu and Dukem in central part of Ethiopia. The objectives of the present study were to isolate and identify *S. aureus, E. coli,* and *Salmonella* from dairy cattle, personnel, and equipment at farms. In addition to this, antimicrobial resistance profiles of the isolates were determined. A total of 607 samples consisting of fresh cow milk (125), fecal sample (211), nasal swab (211), pooled milkers' hand swabs (20), pooled floor swabs (20), and tank milk (20) samples were collected from 20 dairy farms, which included 211 animals. Structured questionnaire was designed and administered to dairy farm owners and dairy food consumers to assess their consumption behavior and antibiotics usage. The samples were examined for the presence of *S. aureus, E. coli,* and *Salmonella* following standard techniques and procedures outlined by the International Organization for Standardization. Subsequently, 62 (15.7%) of *S. aureus* were isolated from 396 of the totals analyzed samples for *S. aureus*. Out of the 62 isolated *S. aureus*, 35/211(16.7%), 19/125(15.2%), 6/20(30%), 2/20(10%), and 0/20(0%) were from nasal swabs, udder milk, bulk milk, pooled hand swab, and floor swabs, respectively. On the other hand, 30 (*7.6*%) of *E. coli* were isolated from 396 of the totals analyzed samples for *E. coli*. Out of the 30 isolated *E. coli*, 17/211(8.1%), 12/125(9.6%), 0/20(0%), 0/20(0%), and 1/20(5%) were from faeces, udder milk, bulk milk, pooled hand swab, and floor swabs, respectively. In line with this, 13 (4.8%) of *Salmonella* were isolated from 271 of the totals analyzed samples for *Salmonella*. Out of the 13 isolated *Salmonella*, 10/211(4.7%), 2/20(10%), 0/20(0%), and 1/20(5%) were from faeces, bulk milk, pooled hand swab, and floor swabs, respectively. Subsequently, 62 of *S. aureus*, 30 of *E. coli,* and 13 of *Salmonella* isolates were subjected to antimicrobial susceptibility testing, and all isolates were resistant to at least one or more antimicrobials tested. Penicillin, methicillin, and trimethoprim/sulfamethoxazole are drugs to which a large proportion of isolated *S. aureus* were highly resistant, which range from 90% to 100%. From 30 tested *E. coli,* they showed (83%) resistance to Tetracycline and 80% to Vancomycin. The resistance level of 13 isolated *Salmonella* was 69% to Nalidixic acid and 54% to Vancomycin. Multiple drug resistance was detected in high (98.4%) for *S. aureus*, (56.7%) for *E. coli,* and (53.9%) for *Salmonella*. High proportion of multiple drug resistant in the dairy farm alerts concern for animal and public health as these drugs are used widely for treatment and prophylaxis in animals and humans.

## 1. Introduction

The safety of dairy products and by-products with respect to food-borne disease is a great concern worldwide. Raw milk may contain pathogenic microorganisms, and it may occasionally play a great role in the transmission of this pathogenic microorganisms to humans [1]. The consumption of raw milk and its derivatives is common in Ethiopia, which is not safe for consumers in a health point of view as it may lead to the transmission of various diseases. Even though milk from a healthy udder contains a few bacteria, it picks up many bacteria from the time it leaves the teat of the animal depending on the hygienic level exercised during milking. These microorganisms are indicators of both manner of handling milk from milking till consumption and the quality of the milk [2–5]. Even ground beef contamination with *Escherichia coli* is usually a result of carcass fecal contamination during the slaughter process. Carcasses are contaminated when they come into contact with soiled hides or intestinal leakage content during dressing and the evisceration processes. A more recent and compelling hypothesis is that when lymph nodes are present in manufacturing beef trimmings, they can be a potential source of *Enterobacteriaceae* contamination of ground beef [6].

The milk and milk products have been a threat to the human since they may contain pathogenic microorganisms. This pathogenic microorganism has been a threat to living. To counter these living threat agents, several measures especially administration of antimicrobials are employed globally. Antibiotics are natural, synthetic, or semisynthetic substances that interfere with the growth or killing of microorganisms, specifically bacteria, and are used to treat or prevent infections in humans and animals. Antibiotics are now an “endangered species” facing extinction due to the worldwide emergence of antimicrobials resistance (AMR) and the void in the development of new therapeutic substances [7, 8]. Nowadays, a global analysis of antimicrobial usage revealed that the worldwide consumption of antimicrobials in food animal production is estimated at ≥57,000 t (1 t = 1,000 kg) and projected a 67% increase in total usage by 2030 to ≥95,000 t [9].

Antimicrobial resistance (AMR) means the ability of a microorganism to survive and reproduce in the presence of antibiotic doses that were previously thought effective against them, which has become an emerging problem globally. In the present time, AMR both in human and veterinary medicine has reached alarming levels in most parts of the world and has been recognized as a significant emerging threat to global public health and food security [10]. Even though global livestock production has been growing rapidly and has moved increasingly where antimicrobial use (AMU) is an integral part of production, their misuse or overuse may produce antimicrobial-resistant bacteria [11]. Moreover, the likelihood that milk and milk products may act as a vehicle for antibiotic-resistant bacterial genes has become a concern to the food industry and a public health issue [12].

Research over the last 40 years has suggested that AMR in east Africa is associated with human-animal contact since they use high levels of antibiotic in small production systems, lack of withdrawal for human consumption of meat and milk products from recently treated animals, and frequent or less prudent AMU. It is reported to be one major reason for failure of treating infectious diseases, which brought the AMR [13]. In The control of drugs from the government authorities and information on the actual rational drug use pertaining to veterinary drug use is very limited, and misuses of drugs are common among the various sectors including veterinary and public health in Ethiopia [14].

Milk production systems can be categorized into urban, semiurban, and rural, based on location, in which around 97% of the annual milk production is accounted for by the traditional milk processing system in Ethiopia [15]. The traditional milk processing materials used are also similar among different areas, which are generally poor in quality of processing, including plastic container, bottle gourd, and clay pot [16, 17]. The fresh milk is sold unpasteurized to the public either directly from small producers, via informal markets or through dairy farmers cooperatives, which has been a great challenge for milk quality control at all levels [18].

Antibiotics have been used in the dairy industry for more than five decades to treat or prevent disease and to increase milk production or improve feed efficiency [19]. Food products such as milk, cheese, yoghurt, and other dairy products have been implicated as potential sources for the transmission of the pathogen to humans [20]. Furthermore, foods, which may be contaminated with antibiotic resistant bacteria, represent ideal vehicles for the transmission of antibiotic resistant strains [21, 22]. So, food is an important vehicle for the transfer of AMR factor to intestinal tract of consumers very efficiently [23, 24]. This further transfer of AMR bacteria to humans via the food chain [21] and from livestock has been well documented, which indicates that livestock and livestock product like milk and meat may serve as reservoirs for human infections [20].

Recently, several antimicrobial-resistant food-borne pathogens have emerged in the food-production chain, which can transmit to and cause infections in humans. *Escherichia coli, Salmonella, and S. aureus* resistance to antimicrobials is one of them and creating trouble to the healthcare system worldwide [25]. Different studies conducted in Ethiopia also revealed the fragmented substantial prevalence as well as antimicrobial susceptibility of *Salmonella*, *E. coli,* and *S. aureus* in veterinary and public health setups [26–32]. As a number of studies show, there is the occurrence of seven major STEC (Shiga toxin producing *E. coli*) serogroups including O157, O145, O103, O121, O111, O45, and O26 [33]. According to the report of Karama et al. [34], only a small number of cattle STEC serotypes that possessed eaeA gene had the highest number of virulence-associated genes, indicative of their high virulence. Moreover, the prevalence of cattle that tested positive for at least one of the six serogroups of STEC across the five farms was variable ranging from 2.9% to 43.4% [33].

However, reports from coinciding study on apparently healthy animals at farm level, personnel, and equipment used in the farms are limited especially in the current study area. Additionally, the risk of consumption of contaminated milk by these resistant bacteria and the selection of resistance in human due to on farm drug misuse or drug residue in milk are not well confirmed. So, the current study was aimed to isolate and identify *E. coli, Salmonella,* and *S. aureus* from different points of dairy farms to determine the level of susceptibility of the isolates related to antimicrobial usage and the public health implications associated with consumption of contaminated milk at selected dairy farms located (Bishuftu and Dukem) in the central part of Ethiopia.

Therefore, the objectives of the current study were as follows:

- To isolate and identify *E. coli, Salmonella,* and *S. aureus* and determine antimicrobial susceptibility profile of the isolated species from selected dairy farms in the study area.

## 2. Material and Methods

### 2.1. Study Area

Bishoftu town is found in east Shewa Zone, Oromia Regional State, located about 45 km south-east of the capital city, Addis Ababa (the area is located at 9°N latitude and 40°E longitude at an altitude of 1850 m above sea level). According to national meteorology agency (NMA) (2016), annual rainfall is 866 mm, of which 84% is in the long rainy season (June to September) with annual minimum and maximum temperatures of 11 and 29°C, respectively. Domestic animals reared in Bishoftu town are 30887cattle, 43138 poultry, 9322 equine, 9294 sheep, and 4753 goats [35].

Dukem town is located at 37 km South East of Addis Ababa along the main road to Adama. Geographically, the study area is located by latitude 8° 45′25″N-8o 50′30″N and longitude 38° 51′55″E–38° 56′5″ E covering a total area of 35.96 km^2^. It is located at an average altitude of 2100 m above sea level. The mean annual rainfall of the area according to 1996 to 2003 year's meteorological data at Bishoftu station is 606.13 mm, and the mean maximum and mean minimum annual temperature of the area are 25.83°C and 11.9°C, respectively. The maximum temperature is during February to May, and the minimum temperature is from mid-October to January. According to 2007 population and housing census, Dukem town has a population of 24,222 [36]

### 2.2. Study Population

The study population was apparently healthy dairy cows, farm equipment used in the storage of milk, environmental sample from floor of the farms, and personnel (hand milkers) in dairy farms located in Bishoftu and Dukem towns.

### 2.3. Study Design

A cross sectional study was conducted to generate the desired data from November 2018 to May 2019.

### 2.4. Sample Size Determination and Sampling Technique

The sample size was calculated using the formula described by Thrusfield and Christley [37] at 5% precision and 20% expected prevalence *E. coli* by Bedasa et al. [38] in Bishoftu town, which was found closer to the selected town. Expected prevalence of *Salmonella* was 4.4% by Dabassa and Bacha [39] and that of *S. aurous* was 16.1% by Beyene et al. [40]. Calculated sample size was 246, 65, and 208, but for this sample, it was increased from 65 to 100 for *Salmonella* only; however, the collected sample was from 211 animals because of unwillingness of farm owners. Generally, the total sample size included in this study was 607 from different sample types.

Twenty dairy farms (proportional allocation of dairy farms from each selected town) were included in this study. The sampling of the farms was employed purposively based on accessibility and willingness of the owners. From total selected farms founded in both towns, all animals found in the farms were included except in two farms, which have large animal size. For those farms of large animal size, proportional animal size with the rest farms was selected randomly and included in the study. From all animals, fecal sample and nasal swab were collected, but milk sample was collected from only lactating animal found during study period. From each farm, pooled tank or bulk milk, pooled hand swabs of milkers, and pooled floor swabs of environmental samples were collected, and *E. coli, Salmonella,* and *S. aureus* were isolated from all samples. From a total of 211 animals, 211 of nasal swaps, 211 feaces samples, and 125 udder milk samples were collected. From all selected farms (20), 20 bulk milk samples, 20 pooled hand swaps, and 20 pooled floor swaps were collected.

*S. aureus* was isolated from nasal swap (211) and udder milk (125) sample. *E. coli* was isolated from feaces sample (211) and udder milk (125). *Salmonella* was also isolated from 211 feaces samples. From all selected dairy farms (20), 20 pooled bulk milk, 20 pooled hand swaps, and 20 pooled floor samples were analyzed for each bacterium (Table 1). So, a total of 396 samples for *E. coli* and *S. aureus* and a total of 271 samples for *Salmonella* were collected and analyzed. Data on antibiotic usage history, disease occurrence, production system, farm hygiene, milk chain and hygiene, and occurrence of drug residue in milk of the farm were collected by using questionnaires.

### 2.5. Sample Collection and Transportation

Sterilized test tubes and cotton-tipped swabs moistened with normal saline were used to collect samples. Aseptic technique was used throughout all sampling and handling procedures by using sterile materials, flaming, and refrigeration. To avoid unpredictable changes, samples were analyzed without delay, and identical samples were analyzed three times in order to confirm the contamination levels. Pure culture was also used for significant studies of microorganisms. Receptacles containing all essential nutrients were free prior sterilized; solution and equipment containing water were autoclaved at 121°C for 15 to 20 min. The media were checked for free of contamination by incubating 5% of the batch at 37°C for 18–24 h, and icebox was used during sample collection, and transportation and the performance of the media were checked using *S. aureus* (ATCC, 25923) for MSA and *E. coli* (ATCC, 25922) for MAC.

Samples from dairy cattle (feacal and nasal swab) and cattle derivative food (milk) were collected aseptically directly from randomly selected healthy dairy cattle in the farm. If diseased animals are presented on the day of sample collection, they were also sampled. Accordingly, approximately 10 ml of milk was collected aseptically from all teats and bulk milk in a sterile test tube. Nasal swabs of animals were collected directly from nose of animals. A pooled swab of milker hands and floor (environment) was collected by using a sterile wooden cotton swab on the surface of material and insert in the 10 ml test tube that contains sterile buffered peptone water (BPW) used as a preenrichment media for 24 hours at 37°C.

The swabs were rotated and rubbed against the sampled surface several times. Faeces were collected directly from the rectum and put into 50 ml containing universal screwed caped bottle. Samples were properly coded based on the collection date, sample source, and sample type. Source of sample was classified as animal, personnel, and equipment. All samples were transported in ice box to Addis Ababa University College of Veterinary Medicine and Agriculture (AAU-CVMA) microbiology laboratory for analysis. Samples were processed separately in preenrichment media and stored at +4°C until processed for the presence of bacteria. Questionnaire survey was also carried out on the dairy farms face to face with farm owners or respondents to assess the antimicrobial usage and public health risks associated with milk consumption resistant bacteria and antibiotic residues in dairy products. All milk samples were processed bacteriologically, and different biochemical tests were performed according to the procedures employed by Quinn et al. [41].

### 2.6. Bacterial Isolation and Identification

#### 2.6.1. *S. aureus*

The isolation and identification of *S. aureus* from different swabs and milk sample were performed at the AAU-CVMA by using techniques recommended by International Organizations for Standardization [42]. Initial culturing was made by streaking 50 *µ*l of each swab-BPW or milk sample on Tryptic soy agar (TSA) with a 5% sheep blood. Plates were incubated at 37°C for 24 hours. Yellow colonies formation with yellow zones after 24 hours of incubation at 37°C on Mannitol Salt Agar (MSA) were appreciated. The presence of *S. aureus* colonies was evaluated using morphological aspects (round, smooth, and white or yellow colonies) and hemolytic pattern (*α*, *β*, double hemolysis (*α* + *β*)) on the surface of blood agar plate and clotted when mixed with 0.5 ml of rabbit plasma.

To get a pure culture, presumed *S. aureus* colonies were further subcultured on nutrient agar plates and incubated at 37°C for 24 hours. In addition to the above colony characteristics, final identification of *S. aureus* was conducted using Gram staining and series of biochemical tests such as coagulase test, catalase test, indole production, methyl red test, Voges-Proskauer reaction, urease production, citrate utilization, and sugar fermentation. Gram-positive cocci in bunched, grape like irregular clusters during gram stain was seen. Bubble production was seen when a loop full of the pure colony was mixed with a drop of 3% hydrogen peroxide on a clean glass slide within a few seconds. Then, the bacterium confirmed as *S. aureus* was subcultured onto nutrient agar and was incubated at 35°C for 20 to 22 h for AM test [43].

#### 2.6.2. *E. coli*

Detection of *E. coli* was carried out according to the protocol of [42] standard from feaces, milk, and swabs samples. Approximately 1 ml of milk/1 g of faeces (homogenized) was suspended into 9 ml of modified Tryptone Soya Broth. Samples were vortexed and incubated overnight at 41°C. After selective enrichment, 50 *µ*l of product was streaked onto MacConkey agar for primary isolation of *E. coli* and incubated aerobically at 37°C for 24 hours. The plates were observed for the growth of *E. coli* (pink colony; lactose fermenter). A single, isolated colony was picked and subcultured on Eosin Methylene Blue (EMB) agar for formation of metallic sheen. Simultaneously, another single colony with similar characteristics was stained with gram stain. The isolate was examined for stain and morphological characteristics using bright-field microscopy. KOH test is then employed to confirm the gram reaction [43].

Suspected colonies of *E. coli* (pinkish color appearance on MacConkey agar and metallic sheen on EMB) were then subcultured onto blood agar to appreciate colony characteristics, and then pure colonies taken from blood agar were inoculated on nutrient agar (nonselective media). Environmental samples were incubated overnight at 41°C after suspended into modified tryptone soya broth (Oxoid) (at 1 : 9 ratios) and subjected to similar tests for bacteriological analysis as fecal and milk samples. Biochemical tests were performed to confirm the *E. coli* using catalase test, Indole Production test, Methyl red-Voges proskaur (MR-VP) test, and Simmon's Citrate test on tryptone broth, MR-VP medium, and Simon citrate agar, respectively [44]. Then, the bacterium confirmed as *E. coli* was subcultured onto nutrient agar and was incubated at 35°C for 20 to 22 h for AM test [43].

#### 2.6.3. *Salmonella*

The isolation and identification of *salmonella* from faeces, different pooled swabs, and bulk or whole milk sample were performed at the AAU-CVMA by using techniques ISO -5659, [44]. And 5 gm of fecal samples was preenriched with 45 ml of BPW at a ratio of 1 : 9, and swabs taken from farm such as floor, hand, and samples of bucket and tank milk were also preenriched with 10 ml BPW and incubated for 24 hrs at 37°C. Then, one ml of the preenriched culture was transferred to 10 ml of Selenite F Broth (SFB) tube, and another 0.1 ml portion was transferred to 10 ml of Rappaport Vassiliadis Soy broth (RVSB) and incubated at 37°C for 24 hrs and 48 hrs, respectively [43].

Finally, one loop of broth culture from the incubated SFB and RVSB was inoculated and incubated on to Xylose Lysine Deoxycholate (XLD) at 37°C for 48 hrs and Salmonella Shigella (S-S) agar at 37°C for 24 hrs. Characteristic *Salmonella* colonies were having a slightly transparent zone of reddish color and a black center on XLD media, and typical *Salmonella* colonies on S-S agar plate cause the color of the medium to be colorless or transparent colony with black center. When suspected colonies are detected, subcultivation of 4 *Salmonella* colonies from XLD and S-S agar on nonselective nutrient agar media plate is done for confirmation by using biochemical tests including Triple sugar iron agar (TSI), Indole test, urease test, Simon's citrate test, and MR-VP test. A typical biochemical reaction on TSI, i.e., alkaline (red) slant, acidic (yellow) but H_2_S and gas production, citrate utilization as a carbon source, Indole and urease negative, M-R positive, and V-P negative were appreciated. Biochemically conformed *Salmonella* were being inoculated in nutrient agar and incubated at 37°C for 24 hrs for AM susceptibility test [45].

### 2.7. Antimicrobial Susceptibility Testing

The antimicrobial susceptibility testing for all bacteria isolates was carried out following the Kirby-Bauer disc diffusion method on Mueller-Hinton agar (Oxoid. CM0337, Basing stokes, England) according to the National Committee for Clinical Laboratory Standards [46]. The isolates were tested with their respective concentration (in brackets) for the following antibiotics; ampicillin (10 *μ*g), amoxicillin/C (20 *μ*g), chloramphenicol (30 *μ*g), gentamycin (10 *μ*g), streptomycin (10 *μ*g), ciprofloxacin (5 *μ*g), kanamycin (30 *μ*g), nalidixic acid (30 *μ*g), cefoxitin (30 *μ*g), and trimethoprim/sulfamethoxazole (23.5 *μ*g) all from Oxoid. From each isolate, some biochemically confirmed well-isolated colonies grown on nutrient agar were transferred into tubes containing 5 ml of Tryptone soya broth (Oxoid, England). The broth culture was incubated at 37°C for 4–6 hr until it achieved the 0.5 McFarland turbidity standards. Sterile cotton swab was dipped into the suspension, and the bacteria were swabbed uniformly over the surface of Mueller-Hinton agar plate (Oxiod, CM 0337, and Basingstoke, England). The plates were held at room temperature for 15–30 min to allow drying. Antibiotic discs with known concentration of antimicrobials were placed, and the plates were incubated for 18–24 hr at 37°C. The bacterial characteristics were the main criteria used to select the antimicrobial agents. Moreover, selection was also based on their mechanisms of action and availability. The diameters of zone of inhibition were recorded to the nearest millimeter and classified as resistant, intermediate, or susceptible according to published interpretive chart [46].

### 2.8. Questionnaire Survey

Structured questionnaires were used to assess the knowledge, attitudes, and practices (KAP) of selected study farms related to antimicrobial usage, residues in products, antimicrobial resistance containments, and risk of milk consumption in the study area. All selected dairy farm owners were included in the study, and data were collected using face-to-face interview and properly recorded questionnaires format. The second questionnaire was used to interview 18 randomly selected consumers on their dairy food consumption habits and the use of antibiotics.

### 2.9. Data Collection, Management, and Analysis

Data describing the presences of isolates in samples were classified, filtered, and coded using Microsoft Excel. The data was then exported to Stat 13 for appropriate statistical analysis. The prevalence of resistance isolates from all samples was determined by using descriptive statistics. Effects were reported as statistically significant by using chi-square test if *P*-value is less than 0.05. Odds ratio and 95% confidence intervals were used to measure the strength of associations.

## 3. Results

### 3.1. Isolation and Identification of *S. aureus, E. coli,* and *Salmonella*

Out of 607 samples collected from 20 dairy farms, 396 samples were analyzed for *S. aureus* and 62 (15.7%) isolates were identified. The occurrence of *S. aureus* in the different sample types, which include nasal swabs, udder milk, pooled hand swabs of personal, milking utensils (bulk milk sample), and pooled floor swabs, was used as indicated in Table 2.

Out of 607 samples collected from 20 dairy farms, 396 samples were analyzed for *E. coli* and 30 (7.6%) isolates were identified. The occurrence of *E. coli* in the different sample types, which include feaces sample, udder milk, pooled hand swabs of personal, milking utensils (bulk milk sample), and pooled floor swabs, was used as indicated in Table 3.

Out of 607 collected samples from 20 dairy farms, 271 samples analyzed for *Salmonella* and 13 (4.8%) isolates were identified. The occurrence of *Salmonella* in the different sample types, which include feaces sample, pooled hand swabs of personal, milking utensils (bulk milk sample), and pooled floor swabs, was used as indicated in Table 4.

### 3.2. Antimicrobial Susceptibility Profiles of *S. aureus, E. coli, and Salmonella* Species

The result of antimicrobial susceptibility test of *S. aureus*, *E. coli,* and *Salmonella* was obtained from the isolated *S. aureus*, *E. coli,* and *Salmonella* 62 isolated *S. aureus*, isolated 30 *E. coli*, and 13 isolated of *Salmonella*), and all isolates were subjected to a panel of 10 antimicrobials (indicated in Table 5). The number of *S. aureus* isolates tested for antimicrobial resistance was 62, and some of the isolated *S. aureus* showed resistance for most of antimicrobials selected but Gentamycin and Ciprofloxacin, which showed 93.6% and 61.3% susceptibility. Penicillin, methicillin, and trimethoprim/sulfamethoxazole were drugs to which a large proportion of *S. aureus* isolates were highly resistant. As indicated in Table 5, most isolates (90% to 100%) were resistant to these three drugs. *S. aureus* isolates were also resistant to Cefoxitin (71%), Tetracycline (66%), Erythromycin (58%), Nitrofurantoin (41%), Streptomycin (40%), and Kanamycin (32%).

Thirty *E. coli* isolates were tested for antimicrobial resistance, and they were found to be resistant to most antimicrobials. Moreover, Gentamycin, Nitrofurantoin, and Ciprofloxacin, which show (90%), (70%), and (63.3%) susceptibility for *E. coli*. From the total of 30 tested isolates, 83% were resistant to Tetracycline, 80% to Vancomycin, 43% to Ceftriaxone, 23% Streptomycin, 20% Cefoxitin, and 17% to Nalidixic acid.

The resistance level of 13 *Salmonella* isolates was 69% to Nalidixic acid, 54% to Vancomycin, 46% to Streptomycin, 46% to Tetracycline, 38% to Ciprofloxacin, and 20% to Cefoxitin. Some other drugs like Gentamycin, Nitrofurantoin, and Ceftriaxone showed 85%, 69%, and 61.5% susceptibility.

The present study revealed that 98.4% (58/62) showed multidrug resistant (MDR) to *S. aureus* isolated from milk of lactating cows, from nasal swabs of animal, bulk milk (milking utensils), and milkers' hands. From all 62 tested isolates of *S. aureus,* one isolate was resistant to all selected drugs expect Gentamycin. Two isolates of *S. aureus* were resistant to 9 drugs except Gentamycin and Ciprofloxacin. Eight isolates were resistant to 8 drugs, and seventeen isolates were resistant to 7 drugs. On the contrary, from all 62 tested isolates of *S. aureus* 58% were susceptible to Gentamycin, and 38% of them were susceptible to Ciprofloxacin.

From a total of 30 tested isolates of *E. coli,* 17 (56.7%) showed MDR. Six isolates of *E. coli* were found to be resistant to Tetracycline, Vancomycin, and Ceftriaxone. On the other hand, most isolates were susceptible to Gentamycin, Ceftriaxone, and Nitrofurantion. From the total of 13 tested isolates of *Salmonella,* 7(56.7%) showed MDR. From all the tested isolates of *Salmonella,* 11 of them were susceptible to Gentamycin and 9 to Nitrofurantion, but all of the five tested isolates were resistant to both Vancomycin and Tetracycline.

Upon comparison of the MDR between sample types, total tested *S. aureus* isolates derived from all four sources, flour, udder milk, and bulk milk sample showed 100% MDR, but from the tested nasal swabs, 97% of them showed MDR. From the total tested *E. coli* isolates derived from flour sample, milk samples, and faeces, 1 isolate of floor sample was tested, and it showed resistance to three of the selected antimicrobials tested. From the 12 tested *E. coli*, those isolated from milk samples showed 5 (41.7%) MDR. On the other hand, all 17 *E. coli* isolated from faeces showed 11 (64.7%) MDR. From the total tested *Salmonella* isolates derived from flour, bulk milk, and faeces samples, 1 tested isolate of flour sample and 2 isolates of bulk milk samples were tested and showed 100% MDR. On the other hand, 10 tested isolates of faeces sample showed 4 (40%) MDR (Table 6).

### 3.3. Questionnaire Survey

The first questioner study was conducted on 20 respondents of farm owners, consisting of 19 males and 1 female. The conducted survey showed that 8 (40%) of respondents completed secondary school, 10 (50%) completed high school, and the rest 2 (10%) were graduated from college. With regard to the location of the farm, all the selected farms were found in the urban location. The dairy cow managed 11 (55%) intensively, 8 (40%) semi-intensively, and 1 (5%) were extensive. All of the respondents used plastic can for milk collection; none of the farms sold their milk to industries, 45% of them sold to small shops, 15% of them sold to cafés and restaurants, and 40% of them to neighbors. Only 20% of farms tested their milk, and the other 80% did not taste their milk, and all of the farms did not pasteurize their milk.

Overall, 51.2% of the dairy herdsmen reported clinical mastitis and dystocia (29.9%). Lameness, left-displaced abomasum, and bloat were also reported. Among herdsmen, 50% reported that they called veterinarians to examine the animals before antimicrobial treatment, 20% of them reported that they bought medicine from veterinary pharmacy and administered the drugs by themselves, 10% of them went to clinic, and 20% of them used traditional medicine. In addition, 45% of the farm owners said that they administered full dose; however, 55% of them stopped the administration of drugs before attaining the complete dose recommended by the clinician. The most commonly used antimicrobials on the dairy farms were 15% ox tetracycline, 40% pen strep, 25% penicillin, and 20% sulfa drug. Regarding the response to knowledge of withdrawal time of drugs they used, 80% of them knew, and 20% of them did not know about drug withdrawal time. None of the farms discarded milk was obtained from a cow in a withdrawal time, 55% the farm gave the milk to calf, 40% of them sold, and 5% of them consumed at home. The other 75% of the respondents said yes when they asked if they knew AMR, and 25% of them replied that they did not know about AMR.

Questions were also developed for the selected farm client consumers. Consumers interviewed on how they consumed the milk they obtained from these farms in this study indicated that, from 18 respondents, 11(61%) of them said they boiled the milk before they consumed. However, 3 (16.7%) used milk for yoghurt or cheese and the other 4 (22%) consumed in both ways. Among them, 53.7% explained their knowledge on the side-effects of raw/undercooked milk consumption.

## 4. Discussions

Antibiotic use drives the evolution of antibiotic resistance [47]. Cow's milk may be contaminated by AMR pathogens from different sources like animals itself, unclean milk containers, the milk handlers, and airborne dust or droplets at the site of production and during processing that presents a health hazard [48]. Over the past years, the dissemination of antimicrobial resistance (AR) in bacteria, including *S. aureus, E. coli,* and *Salmonella,* has increased and posed public health risks. This is best narrated by the multidrug resistant bacterial strains that cause infections, which are difficult to treat. Preserving the effectiveness of existing antibiotics by minimizing the emergence and spread of multidrug resistant microorganisms, which can pass from animal or animal products like milk and meat, are important, so that we can maximize the existing effectiveness of antibiotics [49].

In the present study, the prevalence of *S. aureus* from 396 samples was 62 (15.7%). *S. aureus* was isolated from nearly all sample types with different proportion, except environmental sample (floor swabs), which was (0.0%). This finding is consistent with the study done by Abebe et al. [50] who reported 15.5% in Tigrai, Beyene et al. [40] who reported 16.1% in Addis Ababa, Gizaw and Duguma [51] who reported 17.2% in Addis Ababa, and Osman [52] who reported 17.2% in Egypt. The occurrence of *S. aureus* in the present study was higher than the report from Debre Zeit, which indicated 5% in cottage cheese and 10% in raw milk [53]. Lower prevalence of 6.6% and 10.8% was also reported in India [54] and Brazil [55], respectively. Conversely, a higher prevalence of 40.6% and 42.1%, 40%, 74.5%, and 100% has been reported in Southern [56] and central Ethiopia [57], Morocco [58], India [59], and South Africa [60], respectively.

The prevalence of *E. coli* from 396 samples was 7.6% (30/396); of which 8.1% (17/211), 9.6% (12/125), 0% (0/20), 0% (0/20), and 5% (1/20) were positive from faeces, udder milk, bulk milk, pooled hand swab, and pooled floor swab, respectively. It was not possible to compare the overall prevalence obtained from these five samples types since studies made on *E. coli* isolated from faeces, bulk milk, pooled hand swab, and pooled floor swab were not clearly studied and published in Ethiopia or elsewhere in the world. The current finding is consistent with the studies by Lye et al. [61] and Addo et al. [62] who reported 8.75% and 11.2% from Malaysia and Ghana, respectively. This finding was also lower when we compared it with Abebe et al. [50] finding that showed 23.7% from Tigray, 26% by Farhan et al. [63] from Ethiopia, and 23.3% by Elbagory et al. [64] from Egypt. In addition, the prevalence is far lower when compared to the report of (44%) by Shunda et al. [5] from Mekelle town, Ethiopia, reported 33.9% by Disassa et al. [65] from Ethiopia, report of 69% by Fadaei [66] from Iran, reported 63% by Ali and Abdelgadir [67] from Khartoum, and reported 90.67% Lubote et al. [68] from Tanzania.

In the present study, with regards the prevalence of 4.8% (13/271) of *Salmonella*, 4.7% (10/211), 10% (2/20), 0% (0/20), and 5% (1/20) were positive from faeces, bulk milk, pooled hand swab, and pooled floor swab, respectively. It was also not possible to compare the overall prevalence obtained from these four sample types because studies made on *Salmonella* isolated from faeces, bulk milk, pooled hand swab, and pooled floor swab were not clearly studied in Ethiopia and elsewhere. Overall prevalence of 4.8% *Salmonella* from 271 samples of the present study is not consistent with the finding of Abunna et al. [69] who reported 12.1% in udder milk from Modjo town, Ethiopia, 20% reported by Tadesse and Dabassa [70] from Kersa district of Ethiopia. There is also a report of lower prevalence 3.08% by Addis et al. [71] from central Ethiopia and by Eguale et al. [72] 2.3% *Salmonella* from central Ethiopia.

The difference of the above findings in the present study from previous studies might be attributed to differences in environmental conditions of study site, sample size, or differences in management and hygienic practices. In this study, from the sample type's higher number of *Salmonella*, it was isolated from fecal (4.7%) samples, and the prevalence of *E. coli* in fecal (8.1%) was also high next to milk (9.6%). The prevalence of fecal shedding indicates the possible magnitude of environmental contamination. The result of 5% for both *Salmonella* and *E. coli,* which were isolated from floor swabs, shows some level of floor contamination. Thus, fecal collected from these cows could contaminate the milk, farm equipment's, and personnel handlers. Improper hygienic practices and collecting milk in plastic container were also observed during questioner survey in this study. This may pose risk to the farm personnel and the community at large for infection.

The antimicrobial susceptibility tests carried out in current study indicated that 100% of *S. aureus* were resistant to Penicillin followed by 93% to Methicillin and 91% trimethoprim/sulfamethoxazole. Resistance to penicillin (100%) was more frequently observed than other drugs. This result is comparable with the study by Ateba et al. [60] and Gandhale et al. [73] who reported the resistance of 100% and 91.5% of penicillin, respectively. In contrary, this result was higher than the study by Massawe et al. [74] and Al-Thani and Al-Ali [75] who reported the resistance of 55.5% and 60% of penicillin, respectively. Resistance to erythromycin (58%) observed in this study is consistent with report by [76], who found resistance of 61.5% in Turkey. But the findings are inconsistent with the report by Mohanta and Mazumder [77] and Massawe et al. [74] who found resistance among 20.5% and 29% of isolates, respectively. On the other hand, isolates showed moderately low resistance to Ciprofloxacin and sensitivity to Gentamicin. Resistance of Gram-negative organisms to vancomycin occurs because the barrier is impermeable to this antimicrobial, which is ensured by the outer membrane.

The present study showed that the majority of *E. coli* isolates were resistant to Tetracycline with 83.3%, and Vancomycin with 80%. Resistance to Tetracycline was more frequently observed than other drugs. This result is consistent with the study by [78, 79]. But in Dire Dawa, Ahmed and Van Velkinburgh [80] reported that *E. coli* was susceptible to tetracycline, which is contrary to the results of the present study. *E. coli* isolates were sensitive to Streptomycin. The findings of this study are inconsistent with the study by Hiko et al. [81] and Bekele et al. [78] from Ethiopia and Magwira et al. [82] from Botswana revealed the resistance of *E. coli* mainly to streptomycin. Meanwhile, isolates were sensitive to Gentamicin, Ciprofloxacin, and Nitrofurantoin.

All of the 13 isolates of *Salmonella* from animals and farms were tested against 10 commonly used antibiotics. Resistance rates of isolated *Salmonella* were 70% to Nalidixic acid, 54% to Vancomycin, 46% to Streptomycin, and 46.15% to Tetracycline. In the current study, Nalidixic acid showed a least efficacy against *Salmonella* isolates. In addition, the resistance to Nalidixic acid (70%) is consistent with the prevalence of 80% reported from Ethiopia [70], but slightly inconsistent with the prevalence of 89–92% reported from Kenya [83]. The result for Streptomycin resistance (46%) of *isolated Salmonella* in this study was higher than 13.3% and 25%, which was reported by Addis et al. [71] and Tadesse and Dabassa [70], respectively. The effectiveness of Gentamycin in to isolated *Salmonella* in this study 85% is consistent with the result reported by 73.3% and 75% Addis et al. [71] and Tadesse and Dabassa [70], respectively. But lower than Tesfaw et al. [84] who reported 100%.

The frequency of MAR to three or more antibiotics was observed 98.39% in all most in all isolates of *S. aureus*. The present study has demonstrated the presence of high level of multidrug resistance of isolated *S. aureus,* 98.39% (61/62), especially among commonly used drugs like penicillin and tetracycline. The current study is consistent with Tafa et al. [85], Taddesse et al. [86] and Abera et al. [57] who reported 87.6%, 90% and 94.4% of *S. aureus* resistance to multiple drugs, respectively. But the results of this study were higher than the findings reported in Brazil [87] and Ethiopia [88] where 64.4% and 45.1% of *S. aureus* isolates, respectively, resisted three or more antibiotics. According to this study, penicillin, Methicillin, Erythromycin, and tetracycline were the most frequently observed pattern.

Multidrug resistance analysis of *E. coli* showed that 56.67% of tested isolates were resistant to different combinations of tested antibiotics. This is inconsistent with the report of Bedasa et al. [38], who showed that 92.5% of isolates were multidrug resistant. Moreover, various authors [78, 89, 90] from the country and abroad reported multidrug resistance patterns. In the present study, 53.85% of *Salmonella* isolates were resistant to at least three or more types of antimicrobials (MDR) and compared with the work of Tesfaw et al. [84] who reported 50%, but less than the study of Dabassa and Bacha [39] who report 83.3% also Tadesse and Dabassa [70], who reported 70% for multiple antimicrobial resistance.

This difference of AMR in the present study for all isolated bacteria might be due to small sample sizes for the data, nature of drug, presence of different strain of the bacteria, development of resistant gene, and their low frequency usage of some drugs (Gentamycin) for prevention and control of disease in food animals in the study area. This is a concern because the resistant strain can be transmitted to human by consumption of milk and its products. Furthermore, the consumption of food carrying antibiotic-resistant bacteria can directly or indirectly result in the acquisition of antibiotic-resistant infections [91].

Highest resistance levels observed in the present study might be due to none judicious use of antibiotics in dairy farm level. The result of MDR reflects the uses of those antibiotics in the study area were common, and it shows that *S. aureus, E. coli,* and *salmonella* have been exposed to these drugs. From questioner survey common drug used in dairy farms show 15% Oxytetracycline, 40% Penstrep, 25% Penicillin, and 20% Sulfa drug, which almost all tested isolates of bacteria develop resistance to these drugs. It was noticed that these drugs are widely available from agro-vet distributors and can be purchased easily without any prescription from an authorized facility. Among herdsmen, 20% reported that they buy medicine from veterinary pharmacy; this result showed us farm owners buy drugs without prescriptions at some level. Dairy animal owners as well as consumers involved in the study clearly indicated their use of available antibiotics without prescription and appropriate clinical examination/medical consultation.

Evidence [92, 93] indicates that the global rise of antimicrobial resistance is mainly due to indiscriminate use of drug for treatment of both human and animal diseases. Some of the irrational practices in antibiotics usage in the study areas showed self-administration of drugs without proper clinical examination and cessation of drug usage before complete dose. As indicated, 55% of farm respondents did not administer full dose. There was also poor knowledge of withdrawal time of drugs they used; 20% of them did not know about drug withdrawal time, and none of the farms discard milk obtained from a cow in a withdrawal time. Another possible reason for the observed pattern is antimicrobial-resistant bacteria in raw milk that can colonize the gut if consumed by humans, thus making infections difficult to treat the availability and price of these drugs. From the consumer consumption habit, we learned that people can consume raw/undercooked milk products. This aggravates the situation when geared with the irrational use of drugs as investigated in this study. Although different antibiotic classes of drugs are used in animal health management and in human medicine, the selection of resistance to one drug class may lead to cross-resistance to another [94].

## 5. Conclusion and Recommendations

In the present study, isolation of *S. aureus, E. coli,* and *Salmonella* at dairy farms level showed that dairy cattle and their environment are important sources of milk contamination. This indicates poor hygiene, which is great concern to the consumer's health. The current study clearly indicates that *E. coli* and *Salmonella* isolates shaded from faeces can contaminate the milk, the farm equipment, and personnel, resulting from bad hygienic standards of the farms. Detection of high proportion of multiple antimicrobial resistant isolates (98.4%) for *S. aureus*, (56.7%) *E. coli,* and (53.9%) for *Salmonella* in the dairy farm alerts concern for animal and public health as these drugs is used widely for treatment and prophylaxis of various bacterial infections in animals and humans. It also reveals evidence of the irrational use of antibiotics in the dairy production and raw milk consumer habit. The main reasons for the occurrence of a high number of resistant strains in this study are the use of subtherapeutic level of antibiotics and/or short treatment. Finally, due to the high resistance levels detected in the present study, it is believed that it is necessary to set up permanent resistance surveillance programs in the country.

Based on the above remarks, the following recommendations need to be considered:

- To ensure the quality of raw milk, everyone engaged in milk and dairy production chain should be trained for hygienic practices.
- In order to protect consumers from zoonotic AMR, food safety management programs should be implemented and highly considered.
- Public awareness should be raised on the proper use, handling, and storage of antibiotics and should be prioritized for livestock farmers and other drug users.

## Figures and Tables

**Table 1:** Number of samples for each sample type.

Sample type	Sample size
Nasal swab	211
Feaces sample	211
Udder milk	125
Bulk milk	20
Hand swab	20
Floor swab	20
Total	607

**Table 2:** Isolate of *S. aureus* derived from the different sample types.

No.	Sample type	No. of sample	Positive	Frequency (%)
1	Nasal swaps	211	35	16.7
2	Udder milk	125	19	15.2
3	Bulk milk	20	6	30
4	Pooled hand swabs	20	2	10
5	Pooled floor swabs	20	0	0
6	Over all	396	62	15.7

*X* ^2^ = 7.4714, *P*- value = 0.113.

**Table 3:** Isolate of *E. coli* derived from the different sample types.

No.	Sample type	No. of sample	Positive	Frequency (%)
1	Feaces	211	17	8.1
2	Udder milk	125	12	9.6
3	Bulk milk	20	0	0
4	Pooled hand swabs	20	0	0
5	Pooled floor swabs	20	1	5
6	Overall	396	30	7.6

*X* ^2^ = 4.3915, *P*- value = 0.356.

**Table 4:** Isolate of *Salmonella* derived from the different sample types.

No.	Sample type	No. of sample	Positive	Frequency (%)
1	Feaces	211	10	4.7
2	Bulk milk	20	2	10
3	Pooled hand swabs	20	0	0
4	Pooled floor swabs	20	1	5
5	Overall	271	13	4.8

*X* ^2^ = 2.1966, *P*- value = 0.533. Note. *P* value - Level of significance. Significant when *P*-value ≤0.05.

**Table 5:** Antimicrobial susceptibility profiles of *S. aureus*, *E. coli* and *Salmonella*.

Antibiotics	Species	Susceptible		Intermediate		Resistant	
		No	%	No	%	No	%
Ceftriaxone	*S. aureus*	—	—	—	—	—	—
	*E. coli*	16	53.33	1	3.33	13	43.33
	*Salmonella*	8	61.5	4	30.8	1	7.8
Cefoxitin	*S. aureus*	18	29	—	—	44	71
	*E. coli*	17	56.7	7	23.3	6	20
	*Salmonella*	5	38.5	5	38.5	3	23
Ciprofloxacin	*S. aureus*	38	61.3	17.34	27.4	7	11.3
	*E. coli*	19	63.3	10	33.3	1	3.3
	*Salmonella*	—	—	8	61.5	5	38.5
Methicillin	*S. aureus*	3	4.8	1	1.6	58	93.
Gentamycin	*S. aureus*	58	93.6	—	—	4	6.5
	*E. coli*	27	90	—	—	3	10
	*Salmonella*	11	85	1	7.8	1	7.2
Erythromycin	*S. aureus*	12	19.4	14	22.6	36	58
	*E. coli*	—	—	—	—	—	—
	*Salmonella*	—	—	—	—	—	—
Nalidixic acid	*S. aureus*	—	—	—	—	—	—
	*E. coli*	18	60	7	23.3	5	16.67
	*Salmonella*	3	23	1	7	9	70
Nitrofurantoin	*S. aureus*	28	45.1	8	12.9	26	41.94
	*E. coli*	21	70	6	20	3	10
	*Salmonella*	9	69.2	3	23	1	7.8
Penicillin	*S. aureus*	—	—	—	—	62	100
	*E. coli*	—	—	—	—	—	—
	*Salmonella*	—	—	—	—	—	—
Streptomycin	*S. aureus*	8	12.9	29	46.77	25	40.32
	*E. coli*	14	46.6	9	30	7	23.33
	*Salmonella*	6	46.1	1	7.69	6	46.15
Trimethoprim/sulfamethoxazole	*S. aureus*	4	6.45	1	1.61	57	91.94
	*E. coli*	—	—	—	—	—	—
	*Salmonella*	—	—	—	—	—	—
Tetracycline	*S. aureus*	13	20.9	8	12.90	41	66.13
	*E. coli*	5	16.6	—	—	25	83.3—
	*Salmonella*	7	53.8	—	—	6	46.15
Vancomycin	*S. aureus*	—	—	—	—	—	—
	*E. coli*	5	16.6	1	3.3	24	80
	*Salmonella*	3	23.0	3	23.08	7	53.85
Kanamycin	*S. aureus*	30	48.3	12	19.35	20	32.26
MDR	*S. aureus*	1	1.61	—	—	61	98.39
	*E. coli*	13	43.3	—	—	17	56.67
	*Salmonella*	6	46.1	—	—	7	53.85

**Table 6:** Multidrug Resistance pattern of *S. aureus*, *E. coli,* and *Salmonella* isolates.

Bacteria	Antimicrobial resistance level	Number of isolates	Multidrug Resistance, Percent (%)
*S. aureus*	One	One	1.61(1/62)
	Three	Two	3.23(2/62)
	Four	Seven	11.29(7/62)
	Five	Ten	16.13(10/62)
	Six	Fourteen	22.58(14/62)
	Seven	Seventeen	27.42(17/62)
	Eight	Eight	12.90(8/62)
	Nein	Two	3.23(2/62)
	Ten	One	1.61(1/62)
	Total	Sixty-two	100(62/62)
*E. coli*	One	Three	10.00(3/30)
	Two	Ten	33.33(10/30)
	Three	Eight	26.67(8/30)
	Four	Six	20.00(6/30)
	Five	Two	6.67(2/30)
	Six	One	3.33(1/30)
	Total	Thirty	100(30/30)
*Salmonella*	One	Three	23.08(3/13)
	Two	Three	23.08(3/13)
	Three	One	7.69(1/13)
	Four	Five	38.46(5/13)
	Seven	One	7.69(1/13)
	Total	Thirteen	100(13/13)

## Data Availability

The data used to support the findings of this study are available from the corresponding author upon request.
